# Electrically assisted solution blow spinning of PVDF/TPU nanofibrous mats for air filtration applications

**DOI:** 10.55730/1300-0527.3515

**Published:** 2022-11-16

**Authors:** Andinet Kumella ETICHA, Ali TOPTAŞ, Yasin AKGÜL, Ali KILIÇ

**Affiliations:** 1 Mechanical Engineering, Addis Ababa Institute of Technology, Addis Ababa University, Addis Ababa, Ethiopia; 2 Mechanical Engineering Department, Karabük University, Karabük, Turkey; 3 Temag Labs, Faculty of Textile Technology and Design, İstanbul Technical University, İstanbul, Turkey; 4 Safranbolu Vocational School, Karabük University, Karabük, Turkey; 5 Iron and Steel Institute, Karabük University, Karabük, Turkey; 6 R&D Department, Areka Group LLC, İstanbul, Turkey

**Keywords:** Polyvinylidene fluoride, thermoplastic polyurethane, electrically assisted solution blowing spinning, air filtration applications

## Abstract

In this study, pure polyvinylidene fluoride (PVDF), pure thermoplastic polyurethane (TPU), and PVDF/TPU blend nanofibers (1:3, 2:2, 3:1 ratios) were produced via electrically assisted solution blow spinning for air filtration applications. Scanning electron microscopy (SEM) analysis was conducted to investigate the diameters and morphology of nanofibers. The filtration performance of nanofibrous mats was examined by air filtration test with challenging with 0.26 ± 0.07 μm salt particles. Moreover, the flexibility and strength of the samples were determined via tensile tests. Results showed that pure TPU nanofibers had better mechanical properties, while pure PVDF nanofibers showed better filtration performance. However, 3PVDF/1TPU nanofibrous sample had high filtration efficiency (98.86%) close to pure PVDF (99.85%) and better flexibility (32.80% elongation) compared to pure PVDF (11.64% elongation).

## 1. Introduction

Nowadays, great developments have been seen in the field of air filtration to alleviate the issues of air pollution-related problems. However, producing filters with high filtration efficiency and low-pressure drop is a challenging issue [1]. Currently available commercial air filters include high-efficiency particulate air filters (HEPA), melt-blown filters, and glass filters. Apart from HEPA, these air filter devices have relatively low filtration efficiency due to their large pore sizes [2,3]. HEPA filters had a filtration efficiency of 99.97% and they can filter aerosol particle size of ≥0.3 μm [3,4]. Therefore, researchers focus on polymers to produce high air filtration efficiency nanofibers that have one dimension (diameter) in the 10–1000 nanometer (nm) [5] range to filter fine aerosols. Hence, Poly (vinylidene fluoride) PVDF had become one of the more popular membrane materials [6]. This might be due to the remarkable characteristics of PVDF such as high mechanical strength, thermal stability, chemical resistance, and high hydrophobicity, compared to other commercialized polymeric materials [6–8]. Thus, PVDF has been extensively applied in ultrafiltration and microfiltration and is currently being explored by many researchers as a potential candidate in filtration applications [6].

Han et al. [9], reported the performance of the ultrathin poly (vinylidene fluoride-*co*-trifluoro ethylene) (PVDF-TrFE) nanofibers for high-efficiency PM_1.0_ filtration. Results showed that the electrically activated PVDF-TrFE filter demonstrates a PM_1.0_ filtering efficiency of over ≈88% after polarization, which is further improved to ≈94% after triboelectrification. In another study by Li et al. [4], it was investigated that electrospun PVDF resulted in 99.99% filtration efficiency for 0.26 μm NaCl particles with tree-like nanofiber webs with the basis weight of 1 g/m^2^. Also, the pressure drop was only 124.2 Pa which was comparable to ultra-low penetration air filters (ULPA). Moreover, electrospun PVDF nanofiber filter mediums were fabricated to achieve a high filtration efficiency of 99.901% for 0.4 mm particles as reported by Du et al. [10].

Although PVDF has many advantages over other membrane materials in terms of its high mechanical strength and excellent chemical resistance [6], their poor mechanical properties such as brittleness (low elongation) and low surface tension properties of PVDF-based nanofibers restrict their application for filtration [7,8]. To subdue this concern, different techniques have been done so far such as blending with other polymers, reinforcing with nanomaterials, thermal lamination, self-reinforcing methods, and dip-coating [7]. Among these methods, blending PVDF with other polymers is promising for the new class of materials with improved characteristics [11]. Yardimci et al. [12] studied electrospun polyacrylonitrile (PAN)/PVDF blend nanofibers for air filtration applications. In their study, it was found that optimum conditions to obtain uniform and beadless PAN/PVDF nanofibers were confirmed at 20 wt.% PVDF solution content. In another study by Xiao et al. [13], the phase inversion method was used to produce PVDF/PVA blend nanofiber. The dynamic mechanical analysis (DMA) and infrared attenuated total reflection (FTIR-ATR) exhibited the incompatibility of the PVDF and PVA blend. Hence, the study found that introducing PVA into PVDF worsens the mechanical strength of the blend as compared to pure PVDF. Roche and Yalcinkaya [7] studied about air filtration application of electrospun multilayer polyvinylidene fluoride (PVDF) nanofibrous membranes. The result determined that PVDF membranes had high filtration efficiencies: over 99.00% for PM_2.5_, and PM_0.1_.

On the other hand, thermoplastic polyurethane (TPU) is one of the best candidate polymers to be blended with PVDF due to its characteristics of good abrasion resistance, high mechanical properties, better adhesion, and chemical resistance [11]. The blended TPU/PVDF nanofibers can be fabricated by using different techniques. Electrospinning, solution blowing, electroblowing, centrifugal spinning, and melt blowing are the most commonly used methods [14]. Among all the methods, electrospinning is the most frequently utilized fiber fabrication technique [15]. However, this method had some drawbacks such as low production rate, high consumption of electricity, etc. [16]. Therefore, electrically assisted solution blowing (ESBS) was the best alternative to be used to produce PVDF/TPU nanofiber webs. Since this technique can be easily scaled-up to industry, and its production capacity per nozzle was higher than the more commonly used fiber fabrication method [17,18].

To the best of our knowledge, there is no study on the fabrication of PVDF/TPU nanofibers using an electrically assisted solution blowing technique. Thus, in this study, a novel method of electrically assisted SBS will be used to produce PVDF/TPU blend nanofibrous mats. Then, fabricated nanofibers will be going to be optimized to obtain higher filtration efficiency and better elongation (flexibility) for air filtration purposes.

## 2. Materials and methods

### 2.1. Materials

Thermoplastic polyurethane (TPU, BASF C95) and polyvinylidene fluoride (PVDF, Kynar LBG Powerflex) were dissolved in the mixture of dimethylformamide (DMF, Merck) with >99.8% purity and acetone (Merck) with 99.8% purity at a weight ratio of 7:3.

### 2.2. Preparation of PVDF/TPU nanofibrous mats

**Figure 1:**
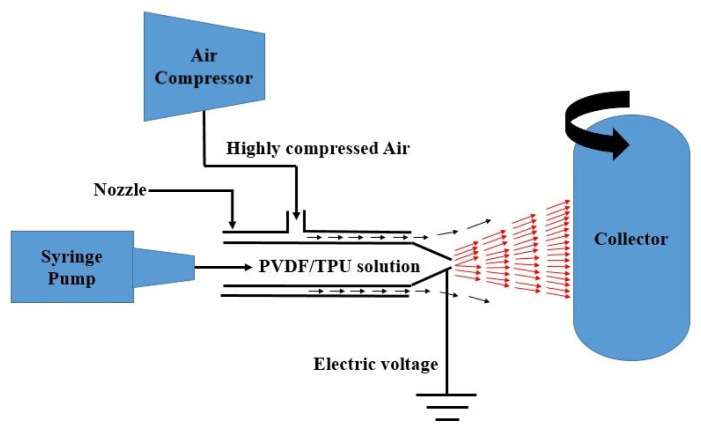
Schematic diagram of electrically assisted solution blowing method.

Pure TPU, pure PVDF, and PVDF/TPU blend solutions were prepared according to Table 1 and the concentration of the solutions was 12 wt.%. Subsequently, nanofibrous mats were prepared by applying an electrically assisted solution blowing (ESBS) fabrication method as schematically represented in Figure 1.

A compressor delivers highly pressurized air at 3 bar to drive the PVDF/TPU solution pumped by a syringe pump through a nozzle needle at a flow rate of 20 mL/h. In addition, to facilitate the solution spinnability an electric 40 kV was connected to the tip of the nozzle. Finally, depending on the solution concentration various nanofibers with different mean diameters were received at the collector which is rotating at 40 rpm after the solvents were evaporated in fiber’s movement from the nozzle tip-to-the collector.

### 2.3. Characterization of PVDF/TPU

The morphology of the nanofibrous mats was investigated using SEM (Zeiss Ultra Plus). The spun fibers were sputtered with a thin layer of gold before observations. Fiber diameters were measured using the ImageJ program. To determine the ductility and strength of samples, the tensile tests were conducted by the Instron 4411 universal testing machine. An automated filter tester (8130A model, TSI Inc.) was used to evaluate the filtration performance pressure drop [ΔP] and filtration efficiency [η] of samples. Here, solid salt particles with a diameter of 0.26 ± 0.07 μm were generated from NaCl solution (2 wt.%). HEPA filters can remove at least 99.97% of particles with a size of 0.3 microns (μm) [19]. Thus, the nanofibrous mats with an effective area of 100 cm^2^ were challenged against the NaCl aerosols with a diameter of 0.26 ± 0.07 at a face velocity of 15.83 cm/s. The filtration efficiencies (η) were calculated by using Equation 1:


(1) $$\eta =1-\frac{\text{Cdown}}{\text{Cup}}$$

where, *C*_down_ is the downstream, and *C*_up_ is the upstream particle concentration. The mathematical expression for the quality factor comprises both filtration efficiency and pressure drop to assess the quality of the filter sample and it is expressed using Equation 2:


(2) $$\text{QF}=-\frac{ln(1-\eta )}{\Delta \text{P}}$$

where, QF is the shorthand form of quality factor, η is the filtration efficiency, and ΔP is the pressure drop [14]. Moreover, the Knudsen number is also used to describe the molecular motions of air molecules close to the fiber surface and can be calculated using Equation 3:


(3) $$\text{Kn}=\frac{2\lambda }{\text{df}}$$

where λ is the mean free path of the gas and the d_f_ is the diameter of the fibers. Considering λ is equal to 65 nm at 298 K and 1 atm [14].

## 3. Results and discussion

### 3.1. Morphological analysis

**Figure 2:**
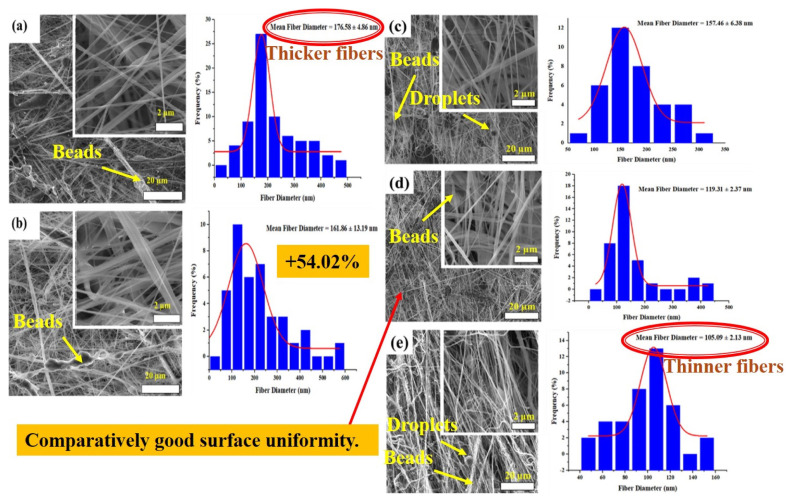
SEM surface morphological images of, (a) TPU, (b) 1PVDF/3TPU, (c) 2PVDF/2TPU, (d) 3PVDF/1TPU, and (e) PVDF.

The morphologies of pure TPU, PVDF, and PVDF/TPU blend nanofibrous mats are indicated in Figure 2. It was observed that beads were noted for all nanofibrous mats. The presence of these beads can be attributed to the turbulent flow of air [20]. Similar SEM nanofiber morphologies were seen in the work of Guo et al. [21]. In their studies, beads coexisted with fibers in pure TPU and blended PET/TPU nanofibers. Also, in the present study droplets were noticed in SEM images of nanofibrous mats. However, 3PVDF/1TPU relatively shows surface uniformity with lower roughness compared to other samples. This is probably having acceptable viscosity compared to all the samples [22]. Also, the incorporation of PVDF into TPU results in a reduction in average fiber diameters. It turned out in Figure 2 that the blended samples (PVDF/TPU) had finer fiber diameter frequencies compared to the sample with pure TPU (Figure 2(a)). The reason for this may be that the viscosity of the solutions increases with the increase in the TPU ratio in the mixtures [19]. While the average fiber diameter of the nanofiber mats obtained with pure TPU was 176 nm as seen in Figure 2a, the average diameter of the fibers of the 1PVDF/3TPU sample was 161 nm as seen in Figure 2b. In addition, as the PVDF ratio in the mixture increased, the average fiber diameters decreased as can be seen in Figure 2c and Figure 2d. Thus, adding 75% by weight TPU to pure PVDF (1PVDF/3TPU) increases the diameter of the PVDF by 54.02%. Thus, very thick fibers were obtained for pure TPU samples. Conversely, finer nanofibrous mats were observed for pure PVDF (Figure 2(e)). This result was in complete agreement with the past study of Hakkak et al. [23]. In their study, it was indicated that decreased fiber diameters were noticed with increased PVDF concentrations.

### 3.2. Filtration performance

**Figure 3:**
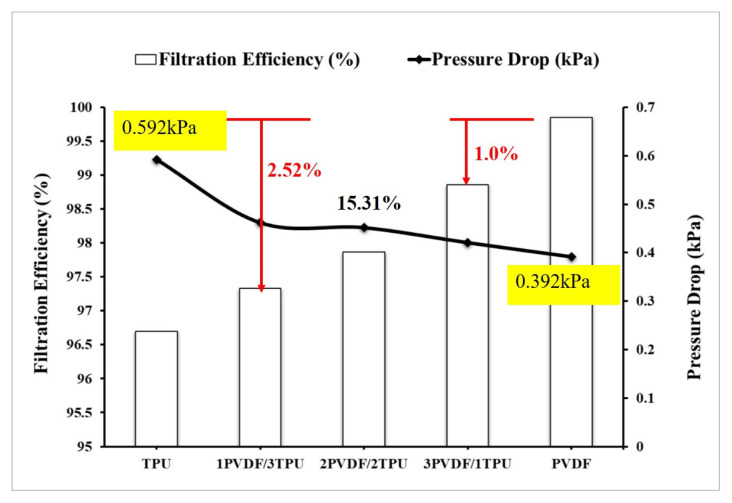
Filtration efficiency and pressure drop of nanofibrous mats.

The air filtration efficiency and pressure drop values of samples are depicted below in Figure 3. It was seen that the highest air filtration efficiency was noticed for pure PVDF nanofibrous mats (99.85%), whereas the least was noted for pure TPU nanofibrous mats (96.7%). This study also showed that the addition of 25 wt.% of TPU into pure PVDF resulted in a decrement in the air filtration efficiency of pure PVDF nearly by 1.0%. Further, adding more TPU (75 wt.%) to pure PVDF (1PVDF/3TPU) results in a remarkable reduction in the filtration efficiency of pure PVDF nearly by 2.52%. This decrement in air filtration efficiency of PVDF with the addition of TPU can be related to the increment of nanofiber’s mean diameter [24].

**Figure 4:**
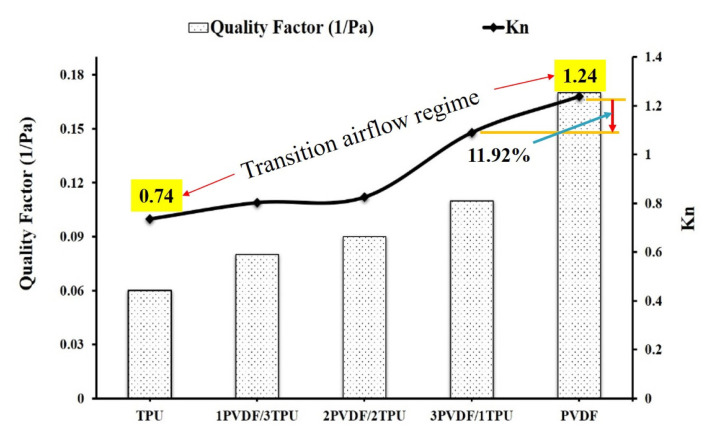
Quality factor and Knudsen numbers (Kn) of nanofibrous mats.

Moreover, pressure drop values of different concentrations of PVDF/TPU blend webs are indicated in Figure 4. Pressure drop has a direct relation with the energy consumption and service life of the nanofibrous mats [20]. TPU nanofiber mat had the highest pressure drop of 592 Pa, but pure PVDF exhibits the lowest with nearly 392 Pa. Also, for 2PVDF/2TPU blend nanofibrous mats addition of 50 wt.% of TPU into pure PVDF results in a pressure drop increment of 15.31%. On the other hand, the Knudsen number is a good indicator for examining the filtration quality of nanofibrous mats. As can be observed from Figure 4, the calculated Knudsen number was in the range of (0.74–1.24), which corresponds to the transition airflow regime [14]. In this regime, air molecules freely move due to the reduction of both drag forces on the nanofiber mat surface and pressure drop. Thus, the addition of TPU onto PVDF causes a reduction in Knudsen number due to an increment in average fiber diameter. Therefore, as the Knudsen number gets lower, it indicates that the air filtration performance of the blend of PVDF/TPU nanofibrous mats has dropped. Thus, air molecules will not freely move but are dragged by fiber surfaces and pressure drop rises. Hence, the incorporation of 25 wt.% TPU into pure PVDF (3PVDF/1TPU) causes a reduction of Knudsen number by 11.92%, whereas its quality factor and filtration efficiency were reduced by 35.3% and 1.0%, respectively. Therefore, when TPU is blended with PVDF in different concentrations, it results in a reduction in Knudsen numbers, so, as this number lowers it indicates that air filtration efficiency will be reduced. Hence, the Knudsen number confirmed the reduction of the air filtration efficiency of the PVDF/TPU blend as compared to pure PVDF. However, 3PVDF/1TPU blend nanofiber had a relatively higher Knudsen number and quality factor compared with other blend samples so its filtration performance was notably good.

The quality factor of nanofibrous mats fabricated by different concentrations of TPU and PVDF is indicated in above Figure 4. It is exhibited in Figure 4 that pure TPU has a lower quality factor as compared with the rest of the nanofibrous samples. Also, the addition of TPU into pure PVDF resulted in a decrement in the quality factor of the blend PVDF/TPU as compared to pure PVDF nanofiber. This is attributed to the increment of pressure drop across the surface of the blend PVDF/TPU nanofibers. Numerically this was confirmed by the relationship that exists between quality factors and pressure drops (inverse relation). Pure PVDF nanofiber has the highest filtration efficiency as it is investigated in Figure 3, so its quality factor is the highest (Figure 4) which is explained by the highest air passing permeability tendency compared with other blended PVDF/TPU samples. Thus, the addition of TPU to pure PVDF reduces the quality factor of the output blend nanofibrous mats.

### 3.3. Mechanical properties

Table 2 shown below indicates the effects of different contents of TPU on deformation, tensile strength, and elongation at the break of PVDF/TPU blends. Here, TPU was blended with PVDF to improve the elongation properties (flexibility) of PVDF. TPU was chosen to blend with PVDF for the fact that TPU shows the great elastomeric characteristic of higher flexibility. Thus, it was investigated that pure TPU exhibits the highest deformation of nearly 18.93 mm with an applied load of around 1.46 N, which was the highest load sustained by the sample compared with the rest of the nanofiber mats. However, PVDF nanofibrous mats show the lowest extension of nearly 3.49 mm at a load of 0.62 N. Furthermore, the addition of 75 wt.% TPU into pure PVDF (1PVDF/3TPU) significantly improves the elongation at break of pure PVDF by 319.07%. Whereas, adding 25 wt.% TPU into pure PVDF (3PVDF/1TPU) improves the elongation at break of pure PVDF by nearly 181.79%. This suggests that when TPU contents decrease in the blend with PVDF, the flexibility of the blend (PVDF/TPU) is also reduced. This is attributed to the existence of weak interfacial adhesion between TPU and PVDF. This study’s results were in agreement with previous studies [8, 25]. In that study, it was investigated that as the content of TPU in blend further reduced to 50 wt.%, there exists weak interfacial adhesion between TPU and PVDF and this led the tensile strength and elongation at the break of the blend to decrease rapidly. In addition, the tensile strength of the PVDF/TPU blend was lower than pure TPU. This might be due to the presence of PVDF in the form of particulate barriers between hydrogen bonding of TPU chains enhancing the adhesion reduction between PVDF and TPU nanofibrous mats [8]. Similar results were also observed in the work of Shehata et al. [26]. In their study, adding higher TPU concentrations (25% and 30%) results in maximum tensile strength of ~7 MPa and breaking strain of ~97%. While pure PVDF and low TPU concentrations (5% and 10%) exhibited low elasticity with tensile strength below 2 MPa and elongation at breakage of 23%.

## 4. Conclusion

In this study, fiber diameters at the nanoscale were produced via electrical-assisted solution blow spinning (ESBS). This suggests that this method is suitable for fabricating ultrafine fibers to filter nanoscale-size aerosols. It was observed that thinner nanofibers (105.09 ± 2.13 nm) were produced using pure PVDF solutions while 100% TPU solution had thicker nanofiber mats (176.58 ± 4.86 nm). The addition of TPU improved the flexibility of PVDF nanofibers but it reduced the air filtration performance. Thus, the study showed that even though the addition of TPU improved the elongation characteristics of PVDF, the air filtration behavior of blend samples was lower as compared with pure PVDF. However, 3PVDF/1TPU which showed high filtration efficiency (98.86%) close to pure PVDF (99.85%) and better flexibility (32.80% elongation) compared to pure PVDF (11.64% elongation) had optimized characteristics. On the other hand, although high air filtration efficiencies of nanofibers mats were obtained via electrically assisted SBS, it was observed that pressure drop values were comparatively high. Therefore, this could affect the service life of the nanofibrous mats and consume high energy, thus further studies can be conducted on the improvement of pressure drop of nanofiber mats via electrically assisted solution blow spinning.

## Figures and Tables

**Table 1:** Polymer solution concentration.

Experiment Code	TPU (wt.%)	PVDF (wt.%)
TPU	100	0
1PVDF/3TPU	75	25
2PVDF/2TPU	50	50
3PVDF/1TPU	25	75
PVDF	0	100

**Table 2:** Tensile test results of PVDF/TPU nanofibrous web samples.

Sample	Force (N)	Deformation (mm)	Tensile strength (MPa)	Elongation (%)
TPU	1.46	18.93	1.99	63.11
1PVDF/3TPU	0.71	14.63	0.99	48.78
2PVDF/2TPU	0.73	11.94	1.03	39.80
3PVDF/1TPU	0.83	9.84	0.96	32.80
PVDF	0.62	3.49	0.20	11.64
